# Mitogenomics suggests a sister relationship of *Relicanthus daphneae* (Cnidaria: Anthozoa: Hexacorallia: *incerti ordinis*) with Actiniaria

**DOI:** 10.1038/s41598-019-54637-6

**Published:** 2019-12-03

**Authors:** Madelyne Xiao, Mercer R. Brugler, Michael B. Broe, Luciana C. Gusmão, Marymegan Daly, Estefanía Rodríguez

**Affiliations:** 1 Department of Invertebrate Zoology, American Museum of Natural History, Central Park West at 79th Street, New York, NY 10024 USA; 2 Biological Sciences Department, NYC College of Technology (CUNY), 285 Jay Street, Brooklyn, NY 11201 USA; 3 Department of Evolution, Ecology and Organismal Biology, The Ohio State University, 300 Aronoff Laboratory, Columbus, OH 43210 USA

**Keywords:** Phylogenetics, Marine biology, Evolutionary biology

## Abstract

*Relicanthus daphneae* (formerly *Boloceroides daphneae*) was first described in 2006 as a giant sea anemone based on morphology. In 2014, its classification was challenged based on molecular data: using five genes, *Relicanthus* was resolved sister to zoanthideans, but with mixed support. To better understand the evolutionary relationship of *Relicanthus* with other early-branching metazoans, we present 15 newly-sequenced sea anemone mitochondrial genomes and a mitogenome-based phylogeny including all major cnidarian groups, sponges, and placozoans. Our phylogenetic reconstruction reveals a moderately supported sister relationship between *Relicanthus* and the Actiniaria. Morphologically, the cnidae of *Relicanthus* has apical flaps, the only existing synapomorphy for sea anemones. Based on both molecular and morphological results, we propose a third suborder (Helenmonae) within the Actiniaria to accommodate *Relicanthus*. Although *Relicanthus* shares the same gene order and content with other available actiniarian mitogenomes, it is clearly distinct at the nucleotide level from anemones within the existing suborders. The phylogenetic position of *Relicanthus* could reflect its association with the periphery of isolated hydrothermal vents, which, although patchy and ephemeral, harbor unique chemosynthetic communities that provide a relatively stable food source to higher trophic levels over long evolutionary timescales. The ability to colonize the deep sea and the periphery of new vent systems may be facilitated by *Relicanthus*’ large and extremely yolky eggs.

## Introduction

Sea anemones (Cnidaria: Anthozoa: Hexacorallia: Actiniaria) are notable for their diversity and ubiquitous distribution in all marine environments. There are ~1,200 described species of these solitary and skeleton-less animals, and despite the relative simplicity of their body plans, anemones show broad morphological and biological diversity: anemones are polyphages and opportunists, feeding on a range of food, from detritus to small vertebrates; they show a variety of reproductive strategies, including sexual and asexual reproduction and different developmental patterns even within the same species; they can also engage in charismatic symbioses with an array of organisms, from unicellular photosynthetic algae (endosymbiosis) to invertebrate and vertebrate partners (e.g. clown fish, hermit crabs).

Among lineages of Hexacorallia, Actiniaria is notable for the diversity of body size, shape, and form, especially in terms of internal anatomy. As a group, Actiniaria is defined not by some shared attribute but by the apparent absence of features that define other orders: for example, as a group, they lack a mineral or protein skeleton, do not form colonies, and do not have labial tentacles. The absence of a clear anatomical synapomorphy and their extreme diversity in morphology have been used to cast doubt on the monophyly of the group^1,2^. Cnidae, and the distinct ultrastructure of the apex (opening point) of the cnida capsules, however, provide a synapomorphy for sea anemones: actiniarians have apical flaps, three triangular plaques that flex outward once the capsules discharge^3^. Rodríguez *et al*.^4^ tested the monophyly of the order using five standard genes for actiniarian phylogenetics (three mitochondrial and two nuclear genes) and proposed a new higher-level classification of the Actiniaria consisting of two suborders (Anenthemonae and Enthemonae). Their results suggested polyphyly of actiniarians, with the former *Boloceroides daphneae* Daly^5^ nesting among other hexacorallian orders, sister to Zoanthidea, but outside the rest of the sea anemones. Because this relationship was not recovered with strong support (maximum likelihood (ML) bootstrap: 62; Bayesian posterior probability: 99), the authors designated a new genus and family for this taxon — currently *Relicanthus daphneae* — and left it as *incerti ordinis*, reflecting the uncertainty of this result^4^. To date, a phylogenetic reconstruction of the order Zoanthidea by Swain^6^ is the only other molecular study to include *Relicanthus*^6^. The ML-based phylogeny utilized a staggered alignment of concatenated nuclear (18S, ITS1, 5.8S, ITS2, 28S) and mitochondrial (12S, 16S, *cox1*) genes, and the tree was rooted with actiniarians from the family Edwardsiidae. *Relicanthus* grouped sister to black corals (order Antipatharia) but with low bootstrap support (49).

*Relicanthus daphneae* was initially described in 2006 as a deep-sea anemone associated with the periphery of hydrothermal vents in the East Pacific Rise (EPR; Daly^5^). Additional specimens morphologically similar and molecularly identical to *R. daphneae* have been collected at the periphery of hydrothermal vents in the East Scotia Ridge (ESR; Scotia Sea, Southern Ocean^7^), and specimens externally resembling *R. daphneae* have been observed in the Galapagos Islands (ER pers. observ.) and at the eastern Clarion-Clipperton Zone in the Pacific Ocean^8^. In addition, early juvenile specimens closely matching DNA signatures (18S and 28S) of *R. daphneae* have been reported growing on plastic debris in the South China Sea^9^. Unusually large for a sea anemone, *R. daphneae* is characterized by long, deciduous, strongly tapering, trailing, pale purple or pink tentacles and a deep purple to purple-red mouth. The lack of features common for most anemones, such as basilar and marginal sphincter musculature, justified (at the time) the position of this animal among the former boloceroidarian anemones^5^. However, molecular data suggested that the lack of features in these taxa is a case of morphological convergence, with the former boloceroidarians having lost these attributes whereas *Relicanthus* never had them^4^. Rodríguez and colleagues^4^ also examined the ultrastructure of the cnidae in *Relicanthus* and found apical flaps on its cnida capsules, resembling those of actiniarians and adding to the confusion of the placement of this taxon.

Anthozoan mtDNA has been shown to evolve significantly slower than other multicellular animals^10–15^. Pratlong *et al*.^16^ compared anthozoan mitochondrial and nuclear sequence data and found that the former is saturated at all phylogenetic levels and thus can lead to spurious phylogenetic inference^16^. The authors found that the level of saturation of all mtDNA genes is similar, but *cob* (cytochrome b) was the least saturated. The ramifications of these findings are that similarity between sequences may not reflect phylogenetic affinity when substitution saturation is elevated^17^. Daly *et al*.^18^ analyzed the phylogenetic signal of two of the three standard mitochondrial genes that are utilized in actiniarian phylogenetic studies and showed that 12S was most effective at recovering well-supported nodes while 16S was significantly less effective^19^. Based on the results of these previous studies, we take two approaches: (1) we concatenate all 13 mitochondrial genes into a single amino acid-based alignment and construct an PhyML and PhyloBayes-based phylogeny, and (2) construct a PhyloBayes-based phylogeny using the *cob* amino acid alignment alone.

Comparison of complete mitogenomes has greatly improved phylogenetic resolution among major groups within the phylum Cnidaria^19–22^ and the class Anthozoa^23–26^. Mitochondrial gene order can be used to supplement evolutionary relationships revealed by phylogenetic studies^27,28^. Using gene order is a potentially powerful tool as rearrangements are generally rare events and are unlikely to exhibit homoplasy^29^; however, reversal to an ancestral state^23^, convergence^30,31^, and major changes within a single genus are known^32^. Many of these issues can be alleviated by simply broadening taxonomic sampling^28,32^. Herein, we sequenced and assembled 15 new sea anemone mitogenomes, including the mitogenome of *Relicanthus daphneae*. This more than doubles the number of currently available anemone mitogenomes on GenBank (n = 14 as of May 2019). We obtained the amino acid-based multiple sequence alignment presented in Kayal *et al*.^20^, which contained 106 taxa, and added the 15 newly-sequenced mitogenomes as well as all newly available mitogenomes from GenBank (included 13 actiniarians, 1 antipatharian and 1 zoanthidean) for a total of 136 taxa in the final dataset. In addition, we reevaluate the cnidae of *Relicanthus* using new terminology that combines several classifications that better reflect the diversity of nematocysts and their phylogenetic patterns of distribution^33^.

## Results

Prior to this study, only 14 anemone mitogenomes were publicly available on GenBank (sizes ranged from 17,446 bp in *Sagartia ornata* to 20,690 bp in *Actinia equina*). By presenting 15 newly-sequenced mitogenomes herein, we have more than doubled the number of available anemone mitogenomes. No new gene orders or unique genes were revealed (Supplementary Tables S1 & S2). However, the mitogenome of the clownfish-hosting *Entacmaea quadricolor* is the longest anemone mitogenome reported to date (at 20,960 bp). The gene order of *R. daphneae* was found to be identical with that of all other published anemone mitogenomes (the only exception is *Alicia sansibarensis*, in which *cox2-nad4-nad6-cob* is located between *nad4L* and *atp8*; Foox *et al*.^25^). Similar to *Metridium senile*, we identified a Group I Intron that codes for a homing endonuclease in the *cox1* gene of *Relicanthus*.

The DNA Walk identified four significant reversals that corresponded to the following regions: the intergenic region between *nad2-rns* and *rns-cox2* and near the middle of *rnl* and *cox1* (Supplementary Fig. S3). No repeats were identified in any of the four regions of interest. A mfold analysis identified a stable stem-loop configuration containing a characteristic T-rich loop in only one of the intergenic regions: i.e., *rns-cox2* (stem-loop diagrams available upon request). Thus, we hypothesize that the origin of replication is located between *rns* and *cox2*; however, we did not identify whether this is the heavy or light origin. This finding supported previous work by Brugler & France^34^ that hypothesized that the origin of replication for the black coral *Chrysopathes formosa* is located between *rns-cob*; this was significant because the only observed difference in gene order between these two closely-related groups was associated with the origin of replication just upstream of *rns*.

**Figure 1:**
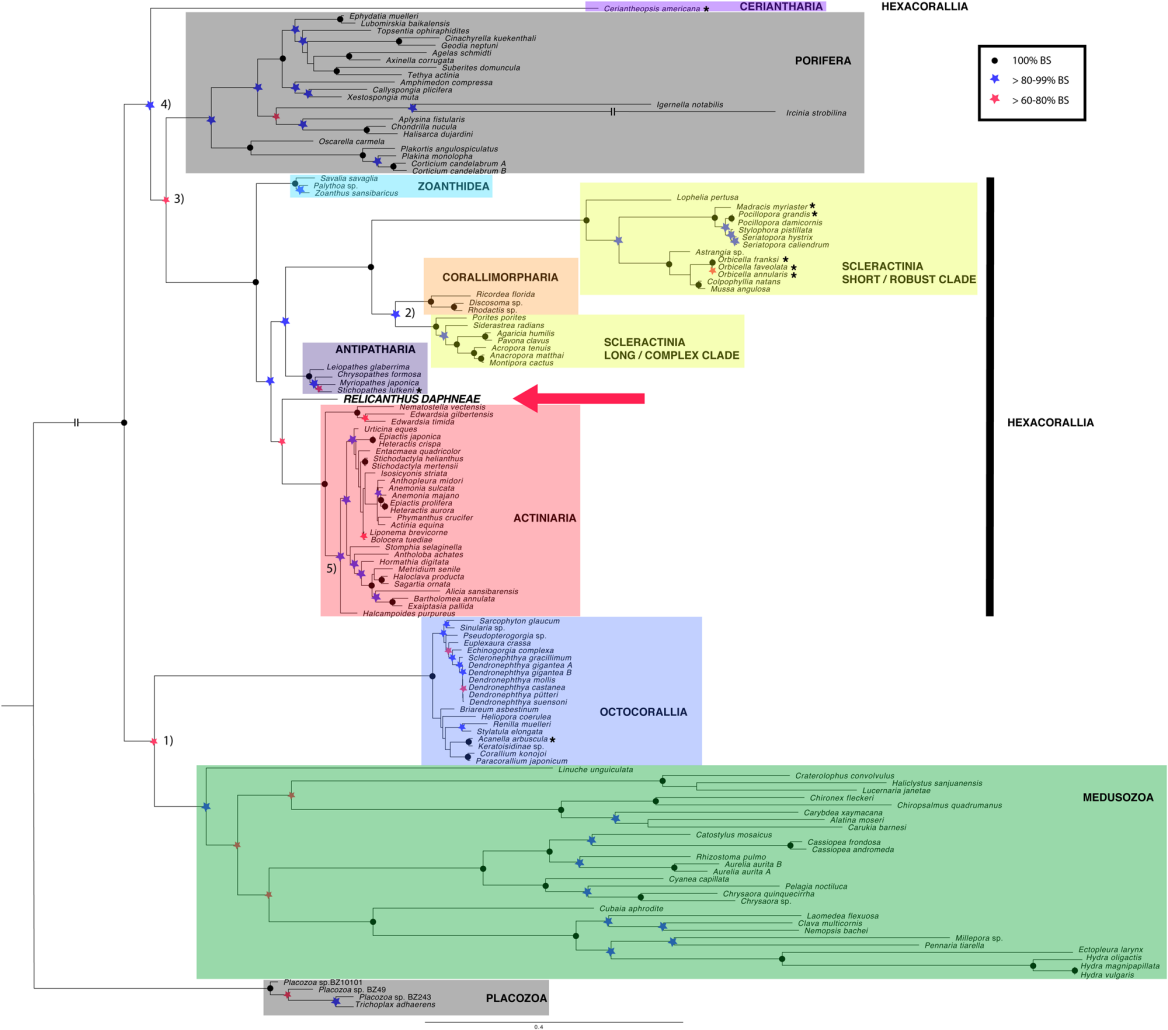
A maximum likelihood phylogenetic reconstruction based on GBlock-edited amino acid sequences of 13 protein-coding genes for 136 taxa (model: LG). The phylogenetic position of *Relicanthus daphneae* is indicated with a red arrow. Node support is based on 1,000 bootstrap replicates (see embedded legend for symbol usage) and the tree is rooted to the Placozoa. For clarity, exceptionally long branches are shortened using hatch marks. Numbers 1–5 next to nodes indicate spurious relationships as discussed in the text. Names of taxa in the tree marked with asterisks have been updated according to WoRMS.

We updated the previous finding of Rodríguez *et al*.^4^ with an ML-based phylogenetic analysis (Fig. 1) based on complete mitogenomes that recovers *R. daphneae* as sister to all other anemones. In addition, we ran a PhyloBayes-based phylogenetic analysis based on complete mitogenomes; however, the chains never converged. Despite this, the topology of both trees was identical. The PhyloBayes analysis based on *cob* alone yielded a polytomy that included *Relicanthus*, the Actiniaria, and a clade comprised of the Antipatharia + Corallimorpharia + Scleractinia.

Bootstrap support for *Relicanthus*’ position in the PhyML tree was 64.7, which was below a previously established cutoff, set at 70, for a supported clade^35^. While the results of Rodríguez *et al*.^4^ called for placement of *Relicanthus* within its own genus and family separated from other actiniarians and a summary reclassification of the order within two suborders, our analysis of the full mitogenome of *Relicanthus* placed it sister to the actiniarians.

Within the Anthozoa, we observed that some of the traditional relationships were resolved but we also recovered unexpected relationships (Fig. 1): octocorals were grouped sister to medusozoans, and corallimorpharians were grouped sister to the long/complex scleractinians. However, both relationships were recovered in former studies^20,36^. In addition, Porifera was resolved within the Cnidaria, and cerianthids (represented by *Ceriantheopsis americana*) were sister to the Porifera + Hexacorallia.

The relationships among sea anemones recovered in our tree roughly corresponded to those recovered by Rodríguez *et al*.^4^. We recovered monophyly of the two current actiniarian suborders (Anenthemonae and Enthemonae) and within Enthemonae, members of the three current superfamilies (Actinostoloidea, Actinioidea and Metridioidea) grouped together, with the exception of *Halcampoides purpureus*. Among the enthemonaean superfamilies, Actinioidea grouped as sister to Actinostoloidea + Metridioidea, a relationship also found by Rodríguez *et al*.^4^, but only when analyzing mitochondrial genes alone.

**Figure 2:**
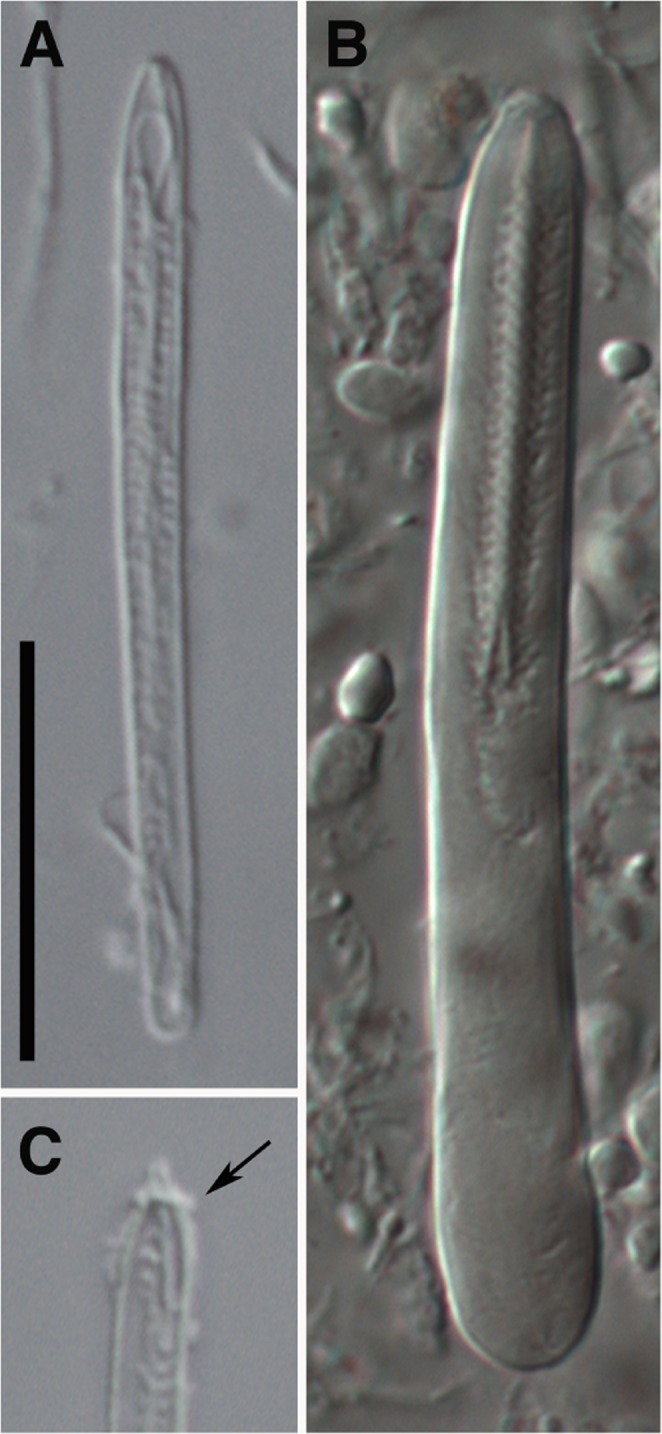
Undischarged nematocyst capsules of *Relicanthus daphneae*. (**A**) Basitrich; (**B**) *p*-mastigophore A (from the actinopharynx); (**C**), detail of the apex of a basitrich showing apical flaps (arrows). Scale bars: A, B, C, 15 micrometers.

Our reevaluation of the cnidae of *Relicanthus* was based on new terminology combining several classifications (see *Material and Methods*), which specifically incorporated means to describe in detail the diversity of mastigophore nematocysts^37^. The cnidae of *R. daphneae* is characterized by the longest spirocysts known within hexacorals, basitrichs with apical flaps, and mastigophore capsules corresponding to *p*-mastigophores A, without spines in the distal tubule (Fig. 2, Rodríguez *et al*.^4^).

Based on the combination of molecular and morphological information, we were able to place the previously *incerti ordinis* Relicanthidae and its single known species, *Relicanthus daphneae*, within the order Actiniaria. We created for it a new suborder, Helenmonae.

### Suborder Helenmonae Daly and Rodríguez

Diagnosis. Actiniaria with well-developed pedal disc but without basilar muscles. No marginal sphincter muscle. Longitudinal ectodermal muscles in column. Deciduous, strongly tapering tentacles, each with sphincter at base. Twenty-four pairs of perfect mesenteries arranged hexamerously. Muscles of mesenteries weak. Cnidom: Gracile spirocysts, basitrichs (with apical flaps), and *p*-mastigophores A.

Included families. Relicanthidae, Rodríguez and Daly 2014 in Rodríguez *et al*.^4^.

Etymology. The name is in honor of the Helen Fellowship program at the American Museum of Natural History, combining it with the Latin word for anemone. The Helen Fellowship, funded by the Helen Gurley Brown Revocable Trust, encourages women to pursue careers in computational science.

Remarks. Although molecular data do not strongly support the affiliation of this taxon within the Actiniaria, the internal anatomy and ultrastructure of its nematocysts (basitrichs with apical flaps and *p*-mastigophores A without spines in the distal tubule) strongly suggest that it belongs within the Actiniaria.

## Discussion

Mitochondrial gene order has been shown to effectively supplement phylogenetic analyses in determining evolutionary relationships^27^. The gene order in *Relicanthus* is the common order for anemone mitogenomes (with one exception: *Alicia sansibarensis*; Foox *et al*.^25^). Based on a maximum composite likelihood analysis (see Supplementary Table S4) of *Relicanthus* with other actiniarians and a one-way ANOVA test of two groups (i.e. genetic distances between *Relicanthus* and actiniarians versus those between actiniarians; see *Materials and Methods*), there is a statistically significant difference (F(1, 56) = 149.12387, p < 0.05; see Supplementary Table S5) between *Relicanthus* and all other actiniarians in terms of the sequence within the mitogenome. Thus, we do not classify *Relicanthus* within either of the two existing actiniarian suborders, but instead establish a new suborder (Helenmonae) of actiniarians containing *Relicanthus* alone. More specifically, *Relicanthus* represents an early-diverging group of anemones without any currently known congenerics; this could explain its relatively slow rate of molecular evolution compared to other actiniarians.

*Relicanthus* is morphologically similar to sea anemones in key features that distinguish it from other hexacorals (i.e. solitary; skeleton-less, no labial tentacles). Similarities in internal organization and mesenterial arrangement (with mono- or dimorphic coupled pairs of mesenteries), and musculature (ectodermal longitudinal musculature in the column — shared only with ceriantharians — lack of basilar and marginal sphincter musculature)^5,38^ are found in both *Relicanthus* and sea anemones. *Relicanthus* is also characterized by deciduous tentacles: the base of the tentacle has a sphincter muscle that allows the animal to autotomize its tentacles^5^. The presence of deciduous tentacles has been reported only within a handful of mostly deep-sea actiniarian genera (e.g. *Bolocera*, *Iosactis*); no other order of hexacorals has this ability, supporting a close relationship of *Relicanthus* with actiniarians. The cnidae of *Relicanthus* also corresponds to that of actiniarians in that their nematocysts have apical flaps, currently the only synapomorphy for the order Actiniaria^4^. In fact, the ultrastructure of the apex of cnida capsules is one aspect of nematocyst morphology that provides phylogenetic information^3^. The discharge of nematocysts is the fastest mechanism in the animal kingdom, occurring in less than three milliseconds^39^. The complexity and strong physiological implications of the opening mechanism suggests homology rather than convergent evolution between the apical flaps of sea anemone nematocysts and those of *Relicanthus*. Although apical flaps are only found in nematocysts of Actiniaria, not all actiniarian nematocysts have them^3^: this is the case of the *p*-mastigophores A (*sensu* Gusmão *et al*.^37^) found in *Relicanthus*, which have a spineless distal tubule similar to those in other actiniarians. *P*-mastigophores A have been hypothesized to be the ancestral type in hexacorals^40,41^, occurring primarily in the mesenterial filaments of all hexacoral orders except Ceriantharia; they are also found in the tentacles of scleractinians, zoanthideans, and some actiniarians as well as in their column and actinopharynx. In the undischarged stage, the basal end tubule of these capsules has a V-shaped notch that is usually more pronounced in non-actiniarians^41^. The lack of spines in the distal tubule of *p*-mastigophores A of actiniarians also distinguishes them from those in other hexacorals, further supporting a close relationship between sea anemones and *Relicanthus*. There is precedence for phylogenetic affinity of other cnidarian orders that share similar cnidae morphologies: scleractinians and corallimorpharians are sister groups, and cnidae between both are virtually identical^40,42,43^. Cnidae morphology is a strong feature even when anatomy is highly divergent, as is the case of myxozoans, whose position within medusozoan cnidarians^44–47^ is further supported by the similar type of apical structure in their polar capsules and those of medusozoans (i.e. operculum)^3,47^. Hence, we consider the similarity of the cnidae of *Relicanthus* and that of actiniarians significant and suggestive of the affinity between *Relicanthus* and the Actiniaria. Thus, evaluation of morphology and cnidae suggest that *Relicanthus* is an anemone, whereas genomic analyses suggest that it belongs to a relatively distinct lineage of anemones (which we reflect by erecting a new suborder of actiniarians), though the support for this placement is moderate.

To date, all documented specimens of *Relicanthus* have been found living in the periphery of hydrothermal vent fields^5,7^, which suggests that *Relicanthus* is simply a background species that inhabits the same depth zone as these food-rich chemosynthetic habitats. Hydrothermal vents are unique environments. They can be separated by tens to thousands of meters (i.e., they are patchily distributed) and the field can remain active for ten to a hundred years^48^ (i.e., they are ephemeral^49^). While active, chemosynthetic communities take advantage of a vent’s geothermal energy and provide a relatively stable food source for organisms at higher trophic levels. Daly^5^ noted that the unusually large size of *Relicanthus* (tentacles >2 m in length and a column diameter of 1 m) would enable it to capture large prey. In addition, the long tentacles of *Relicanthus* are filled with the longest spirocysts known for any hexacoral. Spirocysts are an adhesive kind of cnida exclusive to hexacorals that are commonly associated with prey capture^50^. According to Daly^5^, the diet of *Relicanthus* is unknown, but it is speculated that chemosynthetic bacteria are unlikely to contribute directly to its diet, given *Relicanthus’* distance from active vents (≥100 m). None of the specimens we examined had identifiable food items, but we hypothesize that *Relicanthus* may sustain its large size by opportunistically feeding upon mobile carnivores (e.g., fish, crabs, squat lobsters and octopi) that are entering or leaving the periphery of the vent field to prey upon vent fauna (e.g., tubeworms, clams, mussels, crabs, shrimp, snails, limpets, amphipods, copepods), which are relatively concentrated in space.

Given that *Relicanthus* is a broadly-distributed deep-sea species but is oftentimes found living at the periphery of hydrothermal vents (which mirrors the association between the cosmopolitan stony coral *Lophelia pertusa* and hydrocarbon seeps), *Relicanthus* would need an efficient means of dispersal to relocate to the periphery of another active vent system^51^. According to Daly^5^, *Relicanthus* has the largest eggs found in the order Actiniaria (to 3 mm in diameter of major axis) and the eggs are extremely yolky, suggesting that the larvae are potentially long-lived, thereby facilitating long-distance dispersal. Large egg sizes have also been related to an increase in the chance of fertilization in broadcasting marine invertebrates and deep-sea and polar anthozoans other than *Relicanthus*^52^. To ensure successful dispersal, *Relicanthus* might also use its ability to autotomize its tentacles, a form of asexual reproduction seen in boloceroidarians^53^; however, its ability to disperse asexually is speculative at this point. Deciduous tentacles are also present in other deep-sea anemone genera, but whether they use them as lures, as diversion, or for asexual reproduction is not known. If *Relicanthus* relies on asexual reproduction as its primary means of dispersal, this could account for its relatively slow molecular evolution. However, wide biogeographical distributions with representatives inhabiting chemosynthetic environments (or their periphery) in the north east Pacific and the ESR have also been observed in other invertebrates with no signs of asexual reproduction, such as annelids and asteroideans^7^, suggesting that wide dispersal is possible even without an alternative asexual strategy.

Evolutionary theory predicts that speciation accelerates evolution^54^; that is, the number of nucleotide substitutions between taxa are significantly correlated with the number of speciation events between them. Based on Fig. 1, *Relicanthus* has the shortest branch (i.e., the fewest number of nucleotide substitutions) relative to the outgroup when compared to other members of the order Actiniaria. Additionally, *Relicanthus* does not currently have any closely related sister species; therefore, being a single terminal taxon in our analyses could account for its apparently relatively slow molecular evolution. Thus, it appears that *Relicanthus* is a monospecific genus that, while broadly-distributed in the deep sea, has largely been found inhabiting the periphery of hydrothermal vent systems which may serve as a relatively stable food source over evolutionary time. However, since less than 5% of the deep sea (i.e., depths between 200–10,916 m) has been explored, this preliminary association is subject to revision.

The ML-based phylogenetic reconstruction (Fig. 1) resulted in some unexpected relationships, including (1) a clade of long/complex scleractinians grouping sister to the corallimorpharians (support: 93.9; this result supports the “naked coral” hypothesis as originally shown by Medina *et al*.^36^, (2) the sponges grouping sister to the Hexacorallia (support: 69.5; originally shown by Osigus *et al*.)^55^, (3) the octocorals grouping sister to the Medusozoa (support: 74.5; originally shown by Kayal & Lavrov)^56^, and (4) cerianthids grouping sister to a clade comprised of sponges + hexacorals (support: 81.9; originally shown by Stampar *et al*.)^15^. We consider all of these groupings spurious, reflecting the inadequacy of mitochondrial sequences for reconstructing relationships that diverged hundreds of millions of years ago^16^. We attribute the grouping of *Ceriantheopsis* as sister to the Porifera + Hexacorallia due to either 1) a lack of phylogenetic information (as the *C. americana* mitogenome is represented by only three genes, and two of those genes are partial), and/or 2) long-branch attraction. Though PhyloBayes accounts for long-branch attraction (using the CAT-GTR model), our PhyloBayes analysis did not converge. Lack of convergence could have been due to a lack of informative variability in the dataset (saturation) and/or conflict among the different genes when concatenated. As a result, we are unable to comment on the placement of the cerianthids within our Bayesian inference tree. Stampar *et al*.^22^ recently sequenced the first complete cerianthid mitochondrial genomes (*Isarachnanthus nocturnus* and *Pachycerianthus magnus*) and found that their mitogenomes are composed of five and eight linear chromosomes, respectively. The mitogenome of *I. nocturnus* was also found to be unusually large (80,923 bp).

## Materials and Methods

### DNA extraction

Whole genomic DNA was extracted from tissue and fixed  in 95–100% ethanol using either a 2X-CTAB/chloroform-based DNA extraction protocol^57^ or Qiagen’s Gentra Puregene Tissue Kit, both of which resulted in high molecular weight DNA.

### Sequencing, assembly and annotation of new mitogenomes

For all samples except *Relicanthus*, library preparation and sequencing on an Illumina HiSeq2500 platform was performed by the Genomics Shared Resource at the Ohio State University Comprehensive Cancer Center. For *Relicanthus*, we created a set of three Illumina TruSeq libraries with insert sizes of 180, 400, and 600 bp. Sequencing of 100 bp paired-end reads was conducted on a HiSeq2000 which was located in the EpiGenomics Core at Weill Cornell Medicine. Non-*Relicanthus* reads were assembled using DISCOVAR de novo v. 52488 (Broad Institute, Cambridge, MA, USA) which is optimized for long-read, paired-end Illumina data. In each case, the mitogenome was recovered as a single circular contig. Reads were subsequently mapped back to the DISCOVAR contigs in Geneious 7.1^58^ and assessed for even coverage and agreement. The *Relicanthus* paired-end data were mapped initially to a number of anthozoan mitogenomes in GenBank. The resulting read sets were assembled de novo in Geneious to create seed contigs, which were then extended using the Geneious iterative read mapper employing various parameter settings which balanced extensibility and accuracy until a single circular contig was obtained. Contigs were annotated using MITOS^59^. We carefully examined MITOS scores across loci to rule out false positives and determined open reading frame (ORF) boundaries by transferring homologous gene annotations in Geneious from a representative selection of GenBank anthozoan and medusozoan sequences.

### Creating a multiple sequence alignment

We obtained the amino acid-based multiple sequence alignment presented in Kayal *et al*.^20^, which contained 106 taxa. We then added 15 newly sequenced mitogenomes as well as 15 complete mitogenomes from GenBank that were released after the Kayal *et al*.^20^ study, for a total of 136 taxa in the final dataset including members of Hexacorallia, Octocorallia, Cubozoa, Hydrozoa, Scyphozoa, Staurozoa, Porifera, and Placozoa. The 13 protein-coding genes (*cox1*, *cox2*, *cox3*, *atp6*, *atp8*, *nad1*, *nad2*, *nad3*, *nad4*, *nad4L*, *nad5*, *nad6*, *cob*) were translated separately in AliView v1.18^60^ using translation Table 4 (Mold, Protozoan, and Coelenterate Mitochondrial and Mycoplasma/Spiroplasma) and then aligned separately in MAFFT v7^61^ using the L-INS-i refinement method, with a gap offset value of 0.05. All genes were then concatenated into a single file using MEGA v7^62^. Divergent regions and poorly-aligned positions were identified and filtered using GBlocks v0.91b^63,64^, using the following options for a less stringent selection: allow smaller final blocks; allow gap positions within the final blocks; allow less strict flanking positions. The original dataset consisted of 5,023 sites, but was reduced to 3,390 positions after running GBlocks (shortest sequence: 978 amino acids [*Heliopora coerulea*]; longest sequence: 3,389 amino acids [shared by several taxa]). Using the same settings for less-stringent selection, we ran GBlocks on our *cob* dataset for all taxa; there, 372 sites out of 391 were conserved (95%). Gene tables with the locations of the thirteen genes found across all specimens can be found in Supplemental Table S6.

### Phylogenetic reconstruction using maximum likelihood

We utilized PhyML v3.1^65^ to construct a ML-based phylogenetic reconstruction based on the amino acid alignment. The phylogeny was constructed using the following parameters: model name: LG; proportion of invariable sites and gamma distribution parameter: estimated; amino acid equilibrium frequencies: empirical; tree topology search: best of NNI and SPR; starting tree: BioNJ. Node support was based on 1,000 bootstrap replicates. The phylogeny was rooted with the Placozoa. The analysis took 1,096 hours to complete using an iMac with a 3.8 GHz Intel Core i5 processor and 8 GB 2400 MHz DDR4 memory.

### Phylogenetic reconstruction using bayesian inference

We utilized PhyloBayes v4.1c^66–68^ to construct a Bayesian-inference phylogenetic reconstruction based on the amino acid alignment. We utilized the CAT-GTR model of sequence evolution to negate any potential long-branch attraction. After running PhyloBayes for more than two months (55,030 cycles), the four chains failed to converge (a ‘good’ run has a maxdiff score of less than 0.1; our maxdiff score was never < 1.0). Because the server is a shared resource, we were forced to terminate the run prior to convergence. To visualize the topology of the tree generated at the end of the allotted time period, we sampled every tenth tree after an initial burn-in of 5,000 cycles. We also ran PhyloBayes on the *cob* amino acid-based alignment for all taxa in our dataset (136 taxa; 391 sites); though all four chains converged (maxdiff = 0.0969408), the resulting tree exhibited a polytomy that included *Relicanthus*, the Actiniaria, and a clade comprised of the Antipatharia + Corallimorpharia + Scleractinia.

In addition, we utilized MEGA v7^62^ to conduct a maximum composite likelihood genetic distance analysis (using the nucleotide sequences of all 13 protein-coding genes) of *Relicanthus* and all other actiniarians. We also conducted a one-way ANOVA test of two groups using distance scores resulting from the maximum composite likelihood analysis. We define these two groups as (1) the distances between *Relicanthus* and the actiniarians, and (2) the distances between members of the actiniarians. This test was conducted in order to determine if there is a statistically significant difference between the mitogenomes of *Relicanthus* and other actiniarians.

### Origin of replication

We utilized the DNA Walk program within GraphDNA (virology.uvic.ca) to search the *Relicanthus* mitogenome for abrupt changes in base composition bias that are characteristic of the heavy (OriH) and light (OriL) strand origins of replication. We also searched for repeats using the Tandem Repeats Finder online server (v 4.09) and E-QuickTandem (v 6.6). After locating putative origins of replication, we used the mfold web server to locate stable stem-loop configurations containing characteristic T-rich loops. We utilized default settings for all of the programs noted above^34,69^.

### Morphological analyses

We studied the morphology of two lots of specimens of *Relicanthus daphneae*: One lot from the EPR^5^ (NMNH 1078497: one paratype, collected in 2003) and a second lot of one specimen collected in 2012 during the Antarctic expedition JC80 (ChEsSo) aboard the RSS *James Cook* to the ESR (Southern Ocean). Small pieces of pedal disc and tentacles were fixed in 96% ethanol for DNA analysis and specimens were subsequently fixed in 4% formalin; after fixation, specimens were transferred to ethanol for long term preservation. Two formalin-fixed specimens were examined whole and dissected to check anatomical characters. Cnidae capsules were identified and measured in squash preparations of tissue from tentacles, column, actinopharynx and filaments of one preserved specimen from the ESR (Fig. 2). Preparations were examined using differential interference microscopy (DIC) at 1000x magnification and, except for the rarer types, at least 20 undischarged capsules were measured. Range, mean, and standard deviation were calculated for each type of cnidae; these are not statistically significant but are provided to give an idea of the size distribution of length and width of undischarged capsules. We followed a nematocyst terminology that combines the classification of Weill^70^ modified by Carlgren^71^ — thus differentiating ‘basitrichs’ from ‘*b*-mastigophores’ — with that of Schmidt^40,72,73^ which captures the underlying variation seen in ‘rhabdoids’ (see Gusmão *et al*.^37^ for more details). We include photographs of each type of nematocyst for reliable comparison across terminologies and taxa^37,74^.

## Supplementary information

Supplementary Information

## Data Availability

All newly-sequenced mitochondrial genomes have been deposited in NCBI’s GenBank. Accession numbers are provided in Supplementary Table S6.
