# Supplementary material for: Mitogenomics suggests a sister relationship of Relicanthus daphneae (Cnidaria: Anthozoa: Hexacorallia: incerti ordinis) with Actiniaria
Source: Sci Rep. 2019 Dec 3;9:18182. doi: 10.1038/s41598-019-54637-6 (PMC6890759; doi:10.1038/s41598-019-54637-6)
Supplement: Supplementary file 1 — Supplementary Information [file 41598_2019_54637_MOESM1_ESM.pdf]

GENE	START CODON	START LOC	STOP CODON	STOP LOC	LENGTH (bps)
nad5	atg	0	taa	3551	3551
nad1	atg	966	tag	1949	984
nad3	atg	1959	taa	2315	357
trnW(tga)		3573		3642	
nad2	atg	4009	tag	5148	1140
12s		5327		6319	993
cox2	atg	6366	tag	7115	750
nad4	atg	7182	taa	8657	1476
nad6	ata (ttg?)	8750	taa	9310	561
cytb	atg	9356	tag	10493	1138(?)
trnM(atg)		10623		10693	
16s		11166		12867	1702
cox3	atg	12950	taa	13738	789
cox1_a	ata	13782	tag	14578	797
cox1_b		15505		16354	850
nad4l	atg	16412	taa	16711	300
atp8	atg	16747	tag	16959	213
atp6	atg	17000	taa	17698	699

Title: *R. daphneae* gene table

Caption: List of the start and stop codons, start and stop loci, and lengths of each of the 13 protein-coding genes used in our analysis of Relicanthus daphneae.

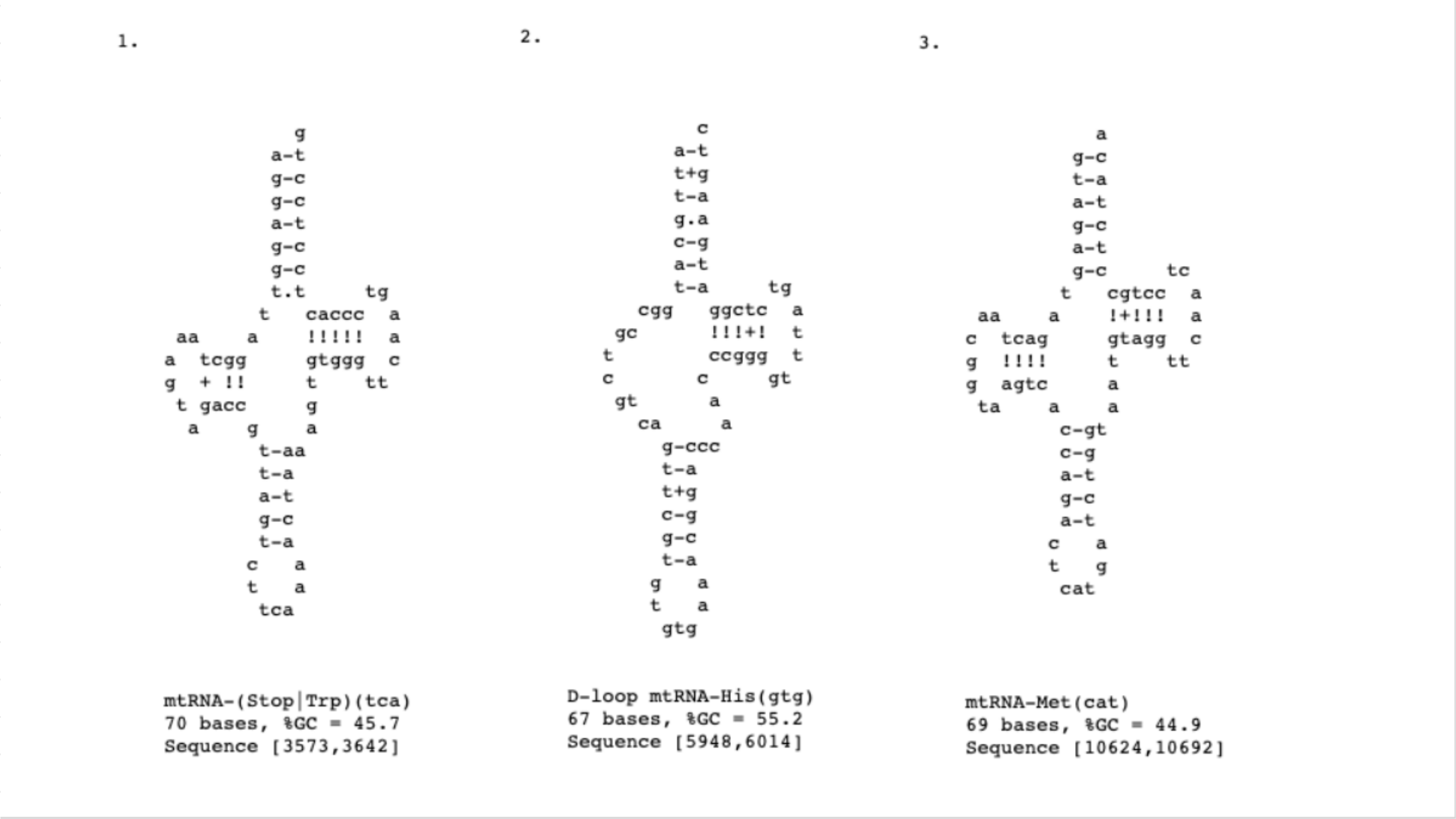

TItle: *R. daphneae* tRNA structures

Caption: Display of all three tRNA structures identified in the mitogenome of Relicanthus.

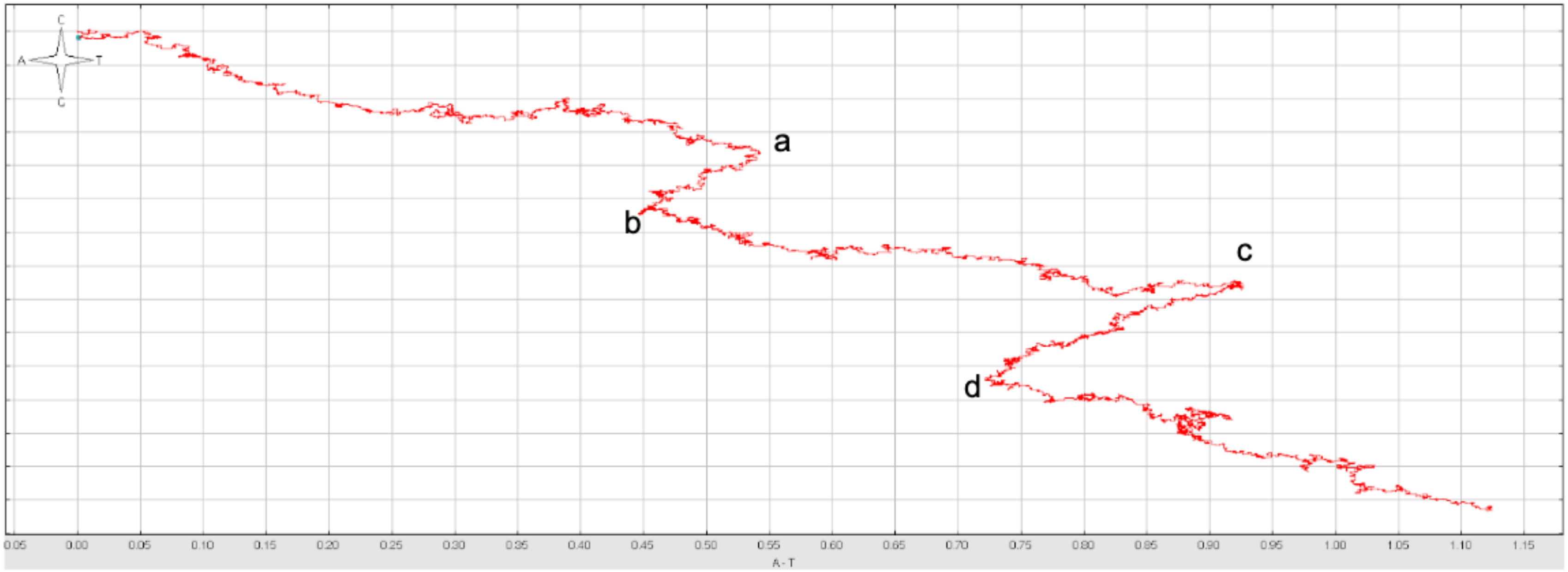

a. 5187 (nad2 ~ 12S)
b. 6303 (12S ~ cox2)
c. 11387 (mid 16S)
d. 14563 (mid cox1)

Title: *R. daphneae* DNA walk

Caption: The DNA walk visualizes the A/T (x-axis) and G/C (y-axis) skew along the Relicanthus mitogenome.

Anemonia sulcat	Anemonia sulcat	Bartholomea ann	Edwardsia gilbert	Edwardsia timida	Entacmaea quad	Epiactis japonica	Epiactis prolifera	Haloclava produc	Heteractis aurora	Heteractis crispa	Liponema brevico	Relicanthus dap	Stichodactyla hel	Stichodactyla me	Stomphia selagir	Antholoba achate	Actinia equina	Phymanthus cruc	Hormathia digitat	Urticina eques	Myriopathes japo	Anemonia majan	Anthopleura mido	Nematostella sp.	Sagartia ornata	Aiptasia pulchella	Halcampoides pu	Alicia sansibaren	Metridium senile	Isosicyonis striata	Bolocera tuediae	Zoanthus sansiba
Bartholomea ann	0.15641718																															
Edwardsia gilbert	0.23105771	0.23768074																														
Edwardsia timida	0.21503271	0.23333707	0.13535791																													
Entacmaea quad	0.06860527	0.16475332	0.2320546	0.20945421																												
Epiactis japonica	0.08227352	0.16200721	0.22729401	0.21040033	0.05596853																											
Epiactis prolifera	0.01865375	0.1576361	0.23190509	0.21655062	0.07506259	0.08737458																										
Haloclava produc	0.12383259	0.0942036	0.22205058	0.21133688	0.13210226	0.12910268	0.12604649																									
Heteractis aurora	0.01865375	0.15788685	0.23190509	0.2162751	0.07506259	0.08737458	0.00038088	0.12628493																								
Heteractis crispa	0.08227352	0.16200721	0.22729401	0.21040033	0.05596853	0	0.08737458	0.12910268	0.08737458																							
Liponema brevico	0.06296631	0.15906229	0.22330706	0.2004746	0.02976382	0.0426384	0.06957615	0.12196331	0.06957615	0.0426384																						
Relicanthus dap	0.2494338	0.27838376	0.27203151	0.27018308	0.24404421	0.25034552	0.25042689	0.25704044	0.25100417	0.25034552	0.24086202																					
Stichodactyla hel	0.0741743	0.1532719	0.21518898	0.20061986	0.05570149	0.05255276	0.07915313	0.12065281	0.07915313	0.05255276	0.03451897	0.23981688																				
Stichodactyla me	0.07269018	0.15442567	0.2179982	0.20176312	0.0522606	0.04985165	0.07864529	0.12032551	0.07864529	0.04985165	0.03108779	0.24235755	0.01153761																			
Stomphia selagir	0.12095512	0.16583847	0.22938015	0.21098989	0.11314524	0.11435664	0.12365801	0.13020989	0.12341995	0.11435664	0.10648543	0.25648048	0.1156236	0.11445928																		
Antholoba achate	0.10899854	0.16233109	0.22869819	0.21666228	0.13617456	0.13275383	0.11072555	0.12754055	0.11072555	0.13275383	0.12777106	0.25720553	0.13266747	0.13269689	0.13748352																	
Actinia equina	0.01817061	0.15554954	0.23052637	0.21278808	0.06735338	0.08059284	0.02368812	0.12271948	0.02368812	0.08059284	0.06056305	0.24786347	0.07456805	0.0729625	0.11831103	0.10738471																
Phymanthus cruc	0.03408533	0.16038986	0.22779718	0.21732245	0.08269933	0.0904292	0.03742484	0.12599999	0.03742484	0.0904292	0.07453982	0.25091436	0.08432326	0.08291437	0.12917294	0.11356152	0.03348286															
Hormathia digitat	0.10913791	0.12826279	0.22437449	0.2115576	0.11567038	0.11646894	0.11193292	0.08385339	0.11239858	0.11646894	0.10928689	0.25356308	0.11053859	0.11081342	0.11710609	0.11935001	0.10745147	0.11407187														
Urticina eques	0.07781471	0.15975055	0.22931734	0.20360769	0.04281998	0.04689817	0.08339582	0.12171727	0.08339582	0.04689817	0.03784382	0.24147656	0.05735058	0.05408916	0.09977366	0.13041714	0.0761208	0.08612814	0.10879918													
Myriopathes japo	0.25945483	0.28202747	0.28663025	0.28114614	0.2591153	0.25519538	0.26126129	0.26534534	0.2615545	0.25519538	0.2517426	0.20460801	0.24772449	0.25203195	0.26335302	0.26047247	0.25989388	0.26387567	0.26233734	0.25227364												
Anemonia majan	0.01973483	0.16042427	0.23187492	0.21652086	0.07494238	0.08715196	0.0099806	0.12809604	0.0099806	0.08715196	0.06947847	0.25124375	0.08080347	0.07941452	0.1248305	0.11085726	0.02408569	0.03894435	0.11343088	0.08372852	0.26004045											
Anthopleura mido	0.01512891	0.15592807	0.23337416	0.21474294	0.06828708	0.08010223	0.02071771	0.12350399	0.02071771	0.08010223	0.06147977	0.25248231	0.07496475	0.07304349	0.11964459	0.10861414	0.01679394	0.03398652	0.10967628	0.07565407	0.26043075	0.02150565										
Nematostella sp.	0.22267225	0.23633945	0.16811146	0.1353788	0.2159616	0.21368843	0.22415648	0.213852	0.22443495	0.21368843	0.20894572	0.27285445	0.20641988	0.20908024	0.21889456	0.2233632	0.22065273	0.2240645	0.217358	0.21178407	0.28940484	0.22803815	0.22276643									
Sagartia ornata	0.12614813	0.09664755	0.22493761	0.21169993	0.13570251	0.13293623	0.12884389	0.02098342	0.12932196	0.13293623	0.12528678	0.26035338	0.12456566	0.12423649	0.13345717	0.13062286	0.12455681	0.12842236	0.08577841	0.12692525	0.26958381	0.13053786	0.12651385	0.21199755								
Aiptasia pulchella	0.1482093	0.03245903	0.23224079	0.22993522	0.15779645	0.15475265	0.14879519	0.08660297	0.14904241	0.15475265	0.15154052	0.26999597	0.14631037	0.14706002	0.15816235	0.15432378	0.14685577	0.15213201	0.12284764	0.15234407	0.27565513	0.1509659	0.14745855	0.23143793	0.08977926							
Halcampoides pu	0.1181344	0.17744107	0.23608871	0.20097042	0.10172595	0.10307996	0.12279241	0.13734836	0.12279241	0.10307996	0.09128584	0.24833912	0.10822001	0.10580489	0.11309138	0.14016741	0.11412141	0.12611232	0.12734033	0.08412789	0.26075669	0.123285	0.11626644	0.21738167	0.14076691	0.17461514						
Alicia sansibaren	0.17666058	0.13475061	0.2686864	0.25790305	0.18483027	0.18477834	0.17685771	0.11522774	0.17737491	0.18477834	0.17935398	0.2922018	0.1789799	0.18052759	0.18345317	0.18190477	0.17562116	0.18264166	0.15028215	0.18126958	0.30091117	0.17773303	0.17659931	0.26173783	0.1177233	0.13054848	0.19091861					
Metridium senile	0.12180056	0.11415234	0.22615083	0.2150575	0.13174069	0.12944304	0.12390567	0.05810406	0.12438062	0.12944304	0.1226561	0.25676269	0.12423512	0.12523427	0.13394395	0.12985571	0.12116274	0.1271926	0.08577436	0.12637951	0.26352944	0.12477401	0.12426184	0.21311547	0.06161001	0.10562561	0.14235879	0.13194104				
Isosicyonis striata	0.06317191	0.1649985	0.23032576	0.20991961	0.03081332	0.05286501	0.06956487	0.13007721	0.06956487	0.05286501	0.02018885	0.244367	0.04922789	0.04572882	0.11529798	0.13612793	0.06098233	0.07644719	0.11582363	0.04756831	0.25982877	0.06912471	0.06189234	0.21471184	0.13417573	0.158719	0.10280225	0.18296524	0.13083445			
Bolocera tuediae	0.06295268	0.15930258	0.22340133	0.20072935	0.02956082	0.0428409	0.06956079	0.12242817	0.06956079	0.0428409	0.00142918	0.24070482	0.03421203	0.03098466	0.10682362	0.12822096	0.06076222	0.07452307	0.10995793	0.03804508	0.25161388	0.06966874	0.06146657	0.20969113	0.12551544	0.15177826	0.09127934	0.17981042	0.12355931	0.02028635		
Zoanthus sansiba	0.2836821	0.30487195	0.31103222	0.30992274	0.29201229	0.29204546	0.28396919	0.28734538	0.28427505	0.29204546	0.28372167	0.23117595	0.28623729	0.28644626	0.29469295	0.29472108	0.28498746	0.28867206	0.28428265	0.28408439	0.25738698	0.28354866	0.28669496	0.31283446	0.28887302	0.3002072	0.28753419	0.31053194	0.28857269	0.28901554	0.28396989	

Title: Maximum composite likelihood analysis on *Relicanthus* and actiniarians

Caption: A maximum composite likelihood analysis was used to generate pairwise distance scores between all actiniarian taxa and *Relicanthus*. Scores generated from comparisons between *Relicanthus* and actiniarians are highlighted in yellow.

Supplementary Table S5

Summary of Data:

	1	2	Total
N	33	33	66
ΣX	5.0201	8.3555	13.3756
Mean	0.1521	0.2532	0.203
ΣX^2	0.9223	2.1237	3.046
St Dev.	0.0704	0.0159	0.0718

Result details

Source	SS	df	MS	
Between treatm	0.1686	1	0.1686	F=64.70714
Within treatmer	0.1667	64	0.0026	
Total	0.3353	65		

Result is significant at $p < 0.05$.

Title: One-way ANOVA test on *Relicanthus* and actiniarians

Caption: Using the distance scores from S4, a one-way ANOVA test was conducted on two test groups -- the first being the scores generated from comparisons of actiniarians to other actiniarians, and the second being the scores generated from comparisons of *Relicanthus* to actiniarians.

Class	Subclass	Order	Suborder	Species	Genbank Access	Museum vouchei	Length (bps)	Complete?	NAD5	NAD1	NAD3	NAD2	COX2	NAD4	NAD6	COB	COX3	COX1	NAD4L	ATP8	ATP6
Anthozoa	Hexacorallia	Actiniaria	E	Anemonia sulcata	MN011067	AMNH	20390	yes	1..717, 2,443..3,558	941..1,924	1,931..2,287	4,014..5,171	6,355..7,095	9,089..10,555	10,560..11,168	11,199..12,347	15,159..15,947	16,199..17,088, 18,142..18,823	19,109..19,408	19,433..19,648	19,682..20,371
			E	Bartholomea annulata	MN066614	AMNH	19615	yes	1..717, 2,472..3,935	940..1,923	1,930..2,286	4,382..5,539	7,237..7,983, 14,	8,846..10,315	10,412..10,927	10,991..12,148	14,523..15,311	15,408..16,300, 17,148..17,847	18,340..18,639	18,663..18,878	18,910..19,599
			A	Edwardsia gilbertensis	MN066615	AMNH	17661	no	1..717, 2,322..3,452	908..1,894	1,896..2,252	3,938..5,077	6,184..6,927	6,932..8,407	8,413..9,021	9,084..10,238	...14,100..14,712	14,774..16,360	16,404..16,703	16,709..16,939	16,951..17,649
			A	Edwardsia timida	##XXXXXX	AMNH	18683	yes	1..717, 2,340..3,471	920..1,903	1,908..2,264	4,212..5,351	8,598..9,344	9,351..10,826	10,832..11,440	11,504..12,658	14,906..15,694	15,793..16,685, 16,686..17,379	17,421..17,720	17,730..17,960	17,972..18,670
			E	Entacmaea quadricolor	MN066616	AMNH	20960	yes	1..717, 2,770..3,885	942..1,925	1,970..2,326	4,603..5,766	6,950..7,690	9,852..11,318	11,323..11,931	11,962..13,110	15,713..16,501	17,196..18,085, 18,939..19,638	19,679..19,978	20,003..20,218	20,252..20,941
			E	Epiactis japonica	MN076184	AMNH	18835	yes	1..717, 2,399..3,553	942..1,925	1,932..2,288	4,468..5,631	6,816..7,556	7,803..9,269	9,274..9,906	9,942..11,090	13,870..14,658	14,829..15,721, 16,575..17,250	17,554..17,853	17,878..18,093	18,127..18,816
			E	Epiactis prolifera	##XXXXXX	AMNH	19754	yes	1..717, 2,455..3,570	941..1,924	1,931..2,287	4,025..5,182	6,367..7,107	9,100..10,566	10,571..11,179	11,210..12,358	14,904..15,692	15,764..16,653, 17,507..18,188	18,473..18,772	18,797..19,012	19,046..19,735
			E	Haloclava producta	MN076185	AMNH	17416	no	1..717, 2,399..3,514	941..1,924	1,931..2,287	3,969..5,126	6,311..7,057	7,063..8,538	8,543..9,151	9,215..10,360	12,748..13,536	13,653..14,545, 15,399..16,098	16,139..16,438	16,463..16,678	16,708..17,397
			E	Heteractis aurora	MN076186	AMNH	19999	yes	1..717, 2,455..3,570	941..1,924	1,931..2,287	4,025..5,182	6,566..7,306	9,299..10,765	10,770..11,378	11,409..12,557	15,149..15,937	16,009..16,898, 17,752..18,433	18,718..19,017	19,042..19,257	19,291..19,980
			E	Heteractis crispa	MN076187	AMNH	18835	yes	1..717, 2,399..3,553	942..1,925	1,932..2,288	4,471..5,631	6,816..7,557	7,803..9,269	9,274..9,906	9,942..11,090	13,870..14,658	14,829..15,721, 16,575..17,250	17,554..17,853	17,878..18,093	18,127..18,816
			E	Liponema brevicorne	MN076188	AMNH	19143	yes	1..717, 2,773..3,888	941..1,924	1,969..2,325	4,599..5,762	6,947..7,693	7,699..9,174	9,179..9,787	9,818..10,966	13,577..14,365	15,024..15,913, 16,767..17,448	17,862..18,161	18,186..18,401	18,435..19,124
			H	Relicanthus daphneae	MK947129	FMNH/NMNH	17727	yes	1..711, 2,433..3,551	966..1,946	1,980..2,315	4,009..5,148	6,420..7,115	7,425..8,657	8,708..9,310	9,528..10,493	12,950..13,738	13,782..14,578, 15,505..16,354	16,430..16,711	16,747..16,959	17,000..17,698
			E	Stichodactyla helianthus	##XXXXXX	AMNH	19551	yes	1..717, 2,399..3,514	942..1,925	1,932..2,288	4,217..5,377	6,536..7,282	7,288..8,763	8,768..9,376	9,407..10,555	13,514..14,302	14,471..15,363, 16,230..16,912	18,273..18,572	18,595..18,810	18,843..19,532
			E	Stichodactyla meretensii	##XXXXXX	AMNH	18849	yes	1..717, 2,399..3,514	942..1,925	1,932..2,288	4,262..5,377	6,536..7,282	7,288..7,810..., ..	8,768..9,376	9,407..10,555	13,315..14,103	14,272..15,164, 16,031..16,712	17,570..17,869	17,893..18,108	18,141..18,830
			E	Stomphia selaginella	##XXXXXX	AMNH	18349	yes	1..717, 2,502..3,617	942..1,925	1,932..2,288	4,202..5,311	6,495..7,259	8,026..9,492	9,497..10,085	10,141..11,286	13,676..14,464	14,612..15,504, 16,334..17,033	17,071..17,370	17,395..17,610	17,642..18,331
			E	Antholoba achates	KR051002	AMNH	17816	yes	6862..7577,9462..10578	7802..8785	8792..9148	11142..12251	13409..14413	14429..15895	15909..16508	16518..17684	2157..2942	3097..3989,4843..5542	5583..5882	5907..6122	6154..6843
			E	Actinia equina	NC_039929.1	AMNH	20690	yes	1..717,2888..4003	863..1924	1931..2287	4374..5750	6935..7675	9664..11130	11135..11743	11770..12921	15664..16452	16687..19112		19733..19948	19982..20671
			E	Phymanthus crucifer (1)	NC_027614.1	AMNH	19727	yes	7187..7903,9815..10930	8127..9110	9117..9473		13861..14601	15988..17454	17468..18067	18095..19246	2262..3050	3276..4165,5031..5712	5865..6164	6190..6405	6439..7128
			E	Hormathia digitata (1)	NC_022471.1	NA	18754	yes	1..717,2399..3514	941..1924	1931..2287	3710..5086	6423..7163	8376..9842	9847..10455	10483..11664	14053..14841	14990..15882,16736..17435	17476..17775	17800..18015	18046..18735
			E	Urticina eques (1)	NC_022469.1	NA	20458	yes	1..717,2399..3514	942..1925	1932..2288	4256..5419	6541..7281	9264..10730	10735..11343	11371..12555	15332..16120	16291..17185,18036..18712	19177..19476	19501..19716	19750..20439
			E	Anemonia majano	NC_037178.1	AMNH	19545	yes	1..717,2397..3512	846..1925	1932..2282	3882..5258	6443..7183	8682..10148	10153..10761	10789..11940	14691..15479	15551..16440,17294..17975	18264..18563	18588..18803	18837..19526
			E	Anthopleura midori	KT989511.1	AMNH	20039	yes	1..716,2431..3547	920..1924	1931..2287	4002..5159	6344..7084	9078..10544	10549..11157	11188..12376	15141..15929	16113..17002,17857..18538	18758..19057	19082..19297	19331..20020
			E	Sagartia ornata (2)	KR051008.1	AMNH	17446	yes	6941..7666,9338..10525	7880..8863	8870..9226	10974..12083	13268..14014	14020..15495	15509..16108	16169..17317	2271..3059	3176..4068,4922..5621	5662..5961	5986..6201	6232..6921
			E	Exaiptasia pallida (2)	NC_022265.1	AMNH	19791	yes	1..717,2448..3902	940..1923	1930..2286	4390..5547	7276..8022	8831..10300	10305..10913	10941..12131	14523..15311	15388..16280,17128..17860	18517..18816	18840..19055	19086..19775
			E	Halcampoides purpureus (2)	NC_027612.1	AMNH	18038	yes	6938..7654,9303..10403	7879..8862	8869..9225	11498..12607	13769..14515	14563..16032	16046..16645	16760..17908	2263..3051	3178..4070,4927..5626	5667..5966	5991..6221	6229..6918
			E	Alicia sansibarensis (2)	NC_027610	AMNH	19575	yes													
			E	Isosicyonis striata (2)	NC_027613.1	AMNH	19001	yes													
			E	Bolocera tuediae (2)	NC_022470.1	NA	19143	yes													
		Zoantharia		Zoanthus sansibaricus (3)	NC_035578.1	NA	20972	yes													
		Antipatharia		Myriopathes japonica (4)	NC_027667.1	NA	17733	yes													

A - Anenthemonae
E - Enthemonae
H - Helenmonae

Title: Table of actiniarian species included in this study, including *Relicanthus*

Caption: List of taxonomic, GenBank accession, voucher location, and gene loci information for taxa included in this study. Abbreviations: AMNH, American Museum of Natural History; FMNH, Field Museum of Natural History; NMNH, National Museum of Natural History. (1), (2), and (3), for information for these taxa see Emblem et al. (2014), Foox et. al (2015), Chi and Johansen (2017), respectively; (4) Unpublished research, only GenBank accession number and information available.
